# Galectin-1-secreting neural stem cells elicit long-term neuroprotection against ischemic brain injury

**DOI:** 10.1038/srep09621

**Published:** 2015-04-10

**Authors:** Jiayin Wang, Jinchao Xia, Feng Zhang, Yejie Shi, Yun Wu, Hongjian Pu, Anthony K. F. Liou, Rehana K. Leak, Xinguang Yu, Ling Chen, Jun Chen

**Affiliations:** 1 Cell Therapy Center, Xuanwu Hospital, Capital Medical University, Beijing 100053, China; 2 Cerebrovascular Center, Henan Provincial People's Hospital, Zhengzhou University, Zhengzhou 450003, China; 3 Center of Cerebrovascular Disease Research, University of Pittsburgh School of Medicine, Pittsburgh, PA 15213, USA; 4 Division of Pharmaceutical Sciences, Mylan School of Pharmacy, Duquesne University, Pittsburgh, PA 15282, USA; 5 Department of Neurosurgery and PLA Institute of Neurosurgery, Chinese PLA General Hospital, Beijing 100853, China; 6 Geriatric Research, Educational and Clinical Center, Veterans Affairs Pittsburgh Health Care System, Pittsburgh, PA 15261, USA

## Abstract

Galectin-1 (gal-1), a special lectin with high affinity to β-galactosides, is implicated in protection against ischemic brain injury. The present study investigated transplantation of gal-1-secreting neural stem cell (s-NSC) into ischemic brains and identified the mechanisms underlying protection. To accomplish this goal, secretory gal-1 was stably overexpressed in NE-4C neural stem cells. Transient cerebral ischemia was induced in mice by middle cerebral artery occlusion for 60 minutes and s-NSCs were injected into the striatum and cortex within 2 hours post-ischemia. Brain infarct volume and neurological performance were assessed up to 28 days post-ischemia. s-NSC transplantation reduced infarct volume, improved sensorimotor and cognitive functions, and provided more robust neuroprotection than non-engineered NSCs or gal-1-overexpressing (but non-secreting) NSCs. White matter injury was also ameliorated in s-NSC-treated stroke mice. Gal-1 modulated microglial function *in vitro*, by attenuating secretion of pro-inflammatory cytokines (TNF-α and nitric oxide) in response to LPS stimulation and enhancing production of anti-inflammatory cytokines (IL-10 and TGF-β). Gal-1 also shifted microglia/macrophage polarization toward the beneficial M2 phenotype *in vivo* by reducing CD16 expression and increasing CD206 expression. In sum, s-NSC transplantation confers robust neuroprotection against cerebral ischemia, probably by alleviating white matter injury and modulating microglial/macrophage function.

Cerebral ischemia is the leading cause of long-term disability and a major cause of morbidity. Current treatment is largely limited to thrombolysis with tissue plasminogen activator (tPA). To avoid the risk of hemorrhagic transformation, the therapeutic window of tPA is limited to 3–4.5 hours after stroke onset. As a result of this limitation, only about 5% of stroke patients have the opportunity for thrombolysis treatment with tPA^1^. Among the numerous efforts to reduce infarction and improve neurological function, stem cell therapy has emerged as a promising approach^2^,^3^,^4^. The mechanisms underlying stem cell therapy are not yet fully understood. Furthermore, the notion that transplanted stem cells work by replacing damaged neural cells has been challenged^5^,^6^. Recent hypotheses have focused on paracrine action, in that stem cells may secrete a battery of factors packaged within extracellular vesicles or secretomes^5^,^6^. This hypothesis has gained considerable traction in recent years because of its implications for the repair or restoration of brain function after injury^5^,^7^,^8^. According to this hypothesis, secretomes contain numerous growth factors, cytokines, metabolites and bioactive lipids, all of which may interact with factors in the surrounding microenvironment^5^,^6^,^9^ and thereby modulate the response to injury or disease.

Recent studies suggest that secretomes contain the regulatory molecule, galectin-1 (gal-1)^10^. Gal-1 is a soluble carbohydrate binding protein with distinct functions. That is, gal-1 is a special lectin, binding specifically to β-galactosides and acting in both the extracellular and intracellular space^11^. In most cases, the extracellular functions of gal-1 require its lectin activity, while its intracellular functions are associated with lectin-independent interactions with other proteins^12^,^13^. In the normal adult brain, gal-1 is only expressed at low levels in neural stem cells (NSCs) of the subventricular zone (SVZ)^14^. However, under pathological conditions such as brain ischemia and experimental allergic encephalomyelitis (EAE), gal-1 levels increase in NSCs and other GFAP-positive cells around the lesions^14^,^15^,^16^. This natural increase in gal-1 after injury might serve as a compensatory mechanism in self-defense, because many neuroprotective effects have been proposed for gal-1. For example, it has been reported that a single injection of gal-1 solution into lateral ventricles stimulates neurogenesis and improves neurological function after focal cerebral ischemia, although it does not reduce infarct volume^17^,^18^. Gal-1 is also a pivotal regulator of neuroinflammation. Recent studies suggested that gal-1 regulates microglial function by preferentially binding to and deactivating the “classically activated” M1 microglia, thereby suppressing the proinflammatory responses associated with CNS demyelinating diseases^15^. Thus far, whether gal-1 also modulates microglial/macrophage polarization in ischemic brains has not been investigated. Furthermore, transplantation of human NSCs promotes functional recovery in gerbils after focal ischemia^19^ and the protective effect is enhanced by overexpression of gal-1 in NSCs (o-NSCs), as indicated by reductions in infarct volume^16^. However, the protection with o-NSCs is not optimal because sensory deficits are only marginally improved compared with the NSC-alone group^19^. The reasons for this deficit are not clear, although it is possible that grafted o-NSCs may not secrete sufficient gal-1 into extracellular space to achieve maximal neuroprotection. We therefore hypothesized that transplantation of gal-1-secreting NSCs (s-NSCs) may afford greater neuroprotection than o-NSCs. To test this hypothesis, we transplanted o-NSCs and s-NSCs into the post-stroke mouse brain, using the transient middle cerebral artery occlusion (MCAO) model. Our results demonstrate that s-NSCs robustly protect the brain over the long-term and to a greater degree than o-NSCs, as indicated by smaller infarcts and improved cognitive and sensorimotor functions. Furthermore, the mechanisms underlying the protective effects of s-NSCs may involve microglia/macrophage polarization and white matter protection.

## Results

### Modified neural stem cells secrete galectin and remain viable 14 days post-ischemia *in vivo*

In this study, we utilized two strategies to improve the protective potential of neural stem cells and examined whether their transplantation into the prosencephalon protects against ischemic injury after MCAO. First, we cloned the NE-4C neural stem cell line stably overexpressing gal-1 (o-NSC), as gal-1 has been reported to prolong the viability of its stem cell host^6^,^11^,^20^. Second, we developed a clone of NE-4C neural stem cells that stably produces and secretes gal-1 (s-NSC), because its role as an extracellular lectin is implicated in the modulation of inflammatory responses^21^,^22^,^23^,^24^. In addition, we also fused a DDK tag to the gal-1 gene in order to distinguish this transgene product from endogenous gal-1. In the characterization of these two clones of neural stem cells, gal-1 overexpression and secretion were visualized by Western blot analysis using anti-galectin-1 and anti-DDK antibodies, respectively. In cell lysates, gal-1 was significantly increased in o-NSC cultures (p = 0.0373 vs. controls), but not in s-NSC cultures (p = 0.0757 vs. controls) compared to empty vector transfected cultures (Supplementary Fig. 1a, lower panel; Supplementary Fig. 1b). DDK immunoreactivity (indicating exogenous gal-1) was also detected only in cell lysates derived from o-NSC and s-NSC cultures. To determine if gal-1 was secreted from the s-NSC cultures, we collected spent media from empty vector transfected, o-NSC, and s-NSC cultures for Western blot analysis. As expected, gal-1 was significantly increased in s-NSC culture media (p = 0.0041 *vs*. controls), but not in o-NSC culture media (p = 0.0771 *vs*. controls) compared to empty vector transfected control cultures (Supplementary Fig. 1a, upper panels; Supplementary Fig. 1b).

Next, we investigated the fate of transplanted neural stem cells in the brain after stroke. Two 3-μl aliquots of s-NSCs (50,000 cells/aliquot) were infused stereotaxically into the cortex and striatum, respectively, at 2 hours after 60 min of MCAO in 2-month-old male C57BL/6j mice. At 7, 14, 21, or 28 days after transplantation, animals were euthanized to examine the presence of transplanted neural stem cells in the brain. At 7 and 14 days after MCAO, clusters of cells in the cortex and striatum were readily detectable near the injection sites (Supplemental Fig. 1c,d,e). Gal-1 and DDK were co-localized in s-NSCs (Supplementary Fig. 1d). However, by 21 or 28 days after MCAO, little immunoreactivity for DDK-gal-1 could be detected in the brain (Supplemental Fig. 1e). Gal-1 levels were also examined by ELISA in striatal extracts at 7, 14, 21, and 28 days after transplantation of o-NSCs or s-NSCs. Consistent with the immunofluorescence results, gal-1 levels were significantly increased in brains transplanted with o-NSCs or s-NSCs compared to vehicle injection at 7 and 14 days post injury (Supplementary Fig. 1f). These results suggest that s-NSCs were viable for about 14 days after transplantation into the post-stroke brain.

It has been reported that the NE-4C neural stem cell line has the capacity to differentiate into neurons or astrocytes^2^,^5^,^11^. We therefore examined the phenotypes of transplanted s-NSCs at 14 days post-injection, by double staining DDK with GFAP (astrocyte marker), NeuN (neuronal marker), or nestin (stem cell marker). Whereas most transplanted s-NSCs showed the expected co-localization with nestin, a small portion of the transplanted cells (<10%) expressed NeuN or GFAP (Supplementary Fig. 1g), suggesting that at this time point the majority of transplanted s-NSC have not differentiated into either neurons or astrocytes.

### Neural stem cell transplantation decreases infarct size and attenuates behavioral deficits after ischemic injury

**Figure 1:**
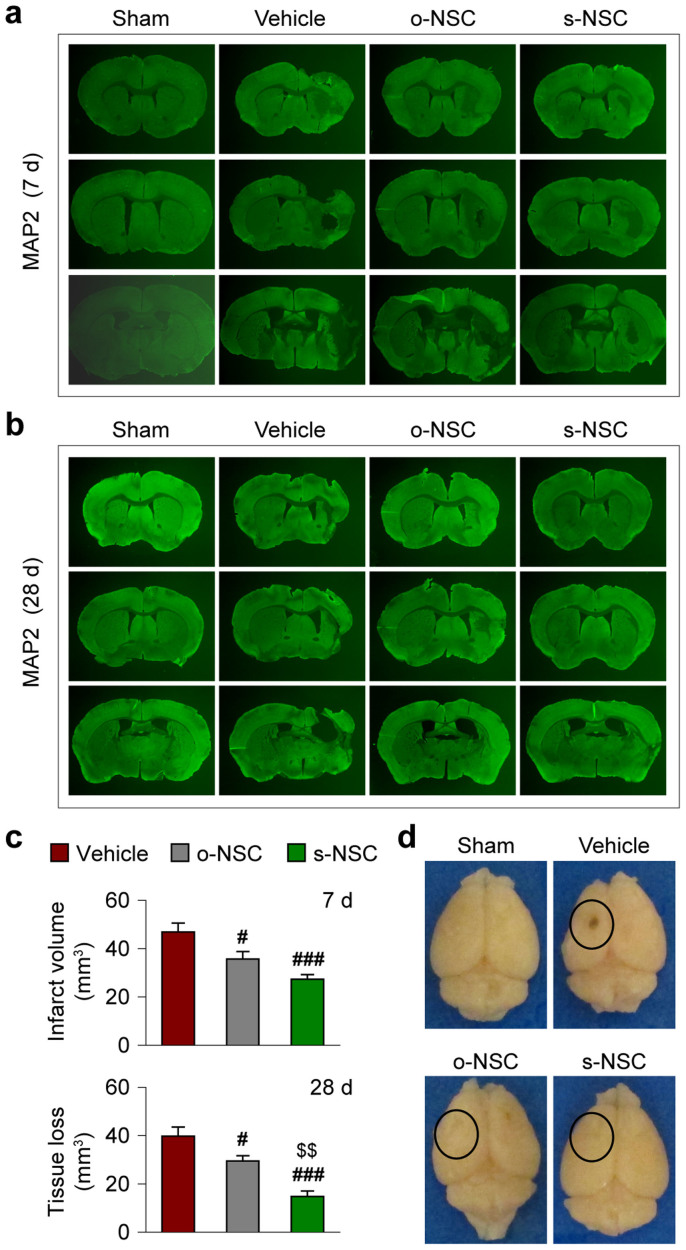
Transplantation of s-NSCs or o-NSCs reduces infarct volume after ischemic injury. Mice received transplantation of o-NSCs or s-NSCs or vehicle injection 2 hours after MCAO. Brain sections were immunostained for MAP2 at 7 and 28 days after MCAO. (a–b) Representative MAP2-stained coronal sections showed smaller infarct or reduced cerebral tissue atrophy at 7 d (a) and 28 d (b) after MCAO in mice receiving transplantation of NSCs. (c) Infarct volume and tissue atrophy were significantly reduced at 7 and 28 days after MCAO, respectively, as compared to vehicle-treated mice. Data are mean ± SEM, n = 7/group, #*p* ≤ 0.05, ##*p* ≤ 0.01 *versus* vehicle; $$*p* ≤ 0.01 *versus* o-NSC. (d) Representative brain images illustrate less brain tissue loss in NSC-treated mice than in vehicle-treated mice.

To investigate the potential protective effects of NSCs against ischemic brain injury, two 3-μl aliquots of s-NSCs, o-NSCs, and non-engineered NSC suspensions (50,000 cells/aliquot) were transplanted into the cortex and striatum, respectively, within 2 hours after MCAO. Regional cortical cerebral blood flow (rCBF) was measured using laser Doppler flowmetry in all mice subjected to MCAO. In 6% of animals rCBF values did not fall below 25% of baseline levels and these mice were therefore excluded from further studies. In addition, approximately 10% of stroke mice died 1–5 days after MCAO and these animals were also excluded. Infarct volume (or tissue atrophy) was measured in coronal sections stained for the neuron-specific marker MAP2 at 7 and 28 days after MCAO by subtracting the volume of viable tissue in the ipsilateral hemisphere from that of the contralateral hemisphere. As shown in Figure 1, tissue atrophy was significantly reduced in mice transplanted with o-NSCs or s-NSCs compared to vehicle-treated mice at both time points. However, transplantation of s-NSCs elicited more robust effects on tissue atrophy than o-NSCs at 28 days after MCAO (Fig. 1c). These results indicate that gal-1 secreting NSCs are more protective over the long term.

**Figure 2:**
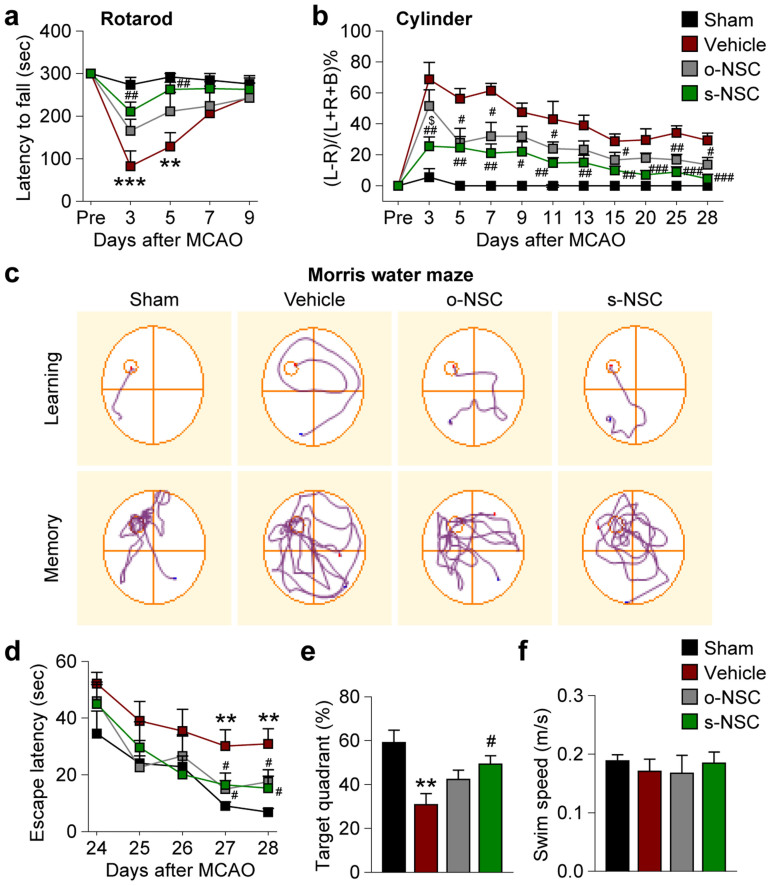
Transplantation of s-NSCs or o-NSCs improves sensorimotor and cognitive functions after ischemic injury. Mice received transplantation of o-NSCs or s-NSCs or vehicle injection 2 hours after MCAO. Three neurobehavioral tests were performed to assess post-stroke neurological deficits up to 28 d after MCAO. (a) The rotarod test showed significantly decreased latency to fall in vehicle-treated mice at 3 and 5 days after MCAO, whereas s-NSC-treated mice exhibited improved performance compared to vehicle-treated stroke mice. (b) In the cylinder test, the number of left, right, or both forepaw contacts were counted, and the performance asymmetry was expressed as (left-right)/(left + right + both) paw use in 5 min. Both s-NSC- and o-NSC-treated mice showed improved performance compared to vehicle-treated mice up to 28 d after ischemia. However, s-NSC-treated mice showed more consistent beneficial effects than o-NSCs-treated mice. (c) Representative images of the swim paths of mice in the Morris water maze test, when the submerged platform was present (learning phase) or after it was removed (memory phase). (d) Time to locate the submerged platform (escape latency) was measured 24–28 d after MCAO or sham surgery. Vehicle-treated mice showed significant deficits at 27 and 28 d after MCAO, whereas both o-NSC and s-NSC groups were significantly improved. (e) Spatial memory (the percentage of time spent in the goal quadrant after the platform was removed) was measured 28 d after MCAO or sham surgery. Only the s-NSC-treated group showed significant improvement compared to the vehicle-treated group after MCAO. (f) Swim speed was comparable among all groups at 27 d after MCAO or sham surgery. All data are mean ± SEM, n = 6–8 mice/group. **p ≤ 0.01 *versus* Sham; #*p* ≤ 0.05 and ##*p* ≤ 0.01 *versus* vehicle; $*p* ≤ 0.05 *versus* o-NSC.

Next, we analyzed the effect of s-NSC and o-NSC transplantation on neurobehavioral deficits induced by MCAO. For assessment of short-term sensorimotor functions, the rotarod test was performed 3–9 days after MCAO. Transplantation of s-NSCs, but not o-NSCs, significantly increased the duration of time on the rod 3 and 5 days after MCAO (Fig. 2a). For long-term assessment of limb use asymmetry, the cylinder test was performed 3–28 days after MCAO. Animals with either s-NSC or o-NSC transplants exhibited more balanced use of both limbs compared to vehicle-treated mice (Fig. 2b). Cognitive performance was assessed by the Morris water maze 24–28 days after surgery. Mice that received either s-NSCs or o-NSCs used less time to locate the hidden platform than vehicle-treated mice, indicating significant improvements in learning capacity (Fig. 2c, upper panels; and Fig. 2d). Furthermore, mice that received s-NSCs, but not o-NSCs, also spent significantly more time in the target quadrant than vehicle-treated mice once the platform was removed (Fig. 2c, lower panels; and Fig. 2e), indicating fewer memory deficits. There was no statistical difference in swim speed between any groups, indicating that the differences in learning and memory could not be attributed to variations in gross motor skills (Fig. 2f). There was no overall difference in learning capacity between mice receiving o-NSCs and those receiving s-NSCs.

### Modified neural stem cells attenuate white matter injury in the corpus callosum and striatum

**Figure 3:**
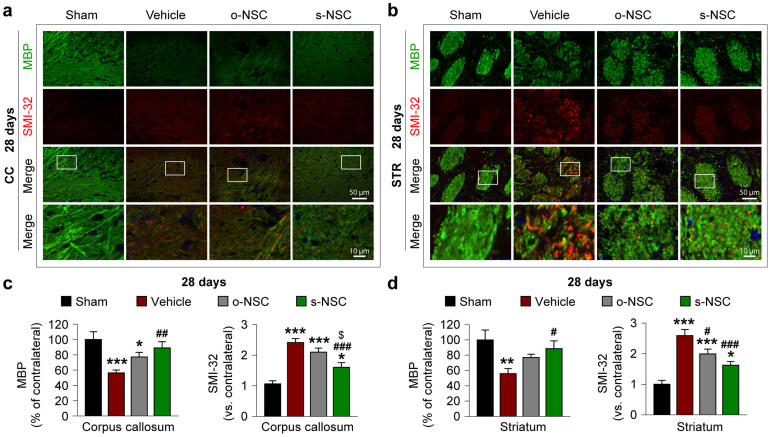
Transplantation of s-NSCs improves white matter integrity 28 days after ischemia. Mice received transplantation of o-NSCs or s-NSCs 2 hours after MCAO. Brain sections were dual-stained for myelin basic protein (MBP) and non-phosphorylated neurofilament H (SMI-32) on day 28 after ischemia. (a–b) Representative immunofluorescent images of MBP and SMI-32 staining in the corpus callosum (a) and striatum (b) after sham surgery or MCAO followed by transplantation of vehicle, o-NSCs, or s-NSCs. (c–d) Quantification of MBP and SMI-32 immunofluorescence in the corpus callosum (c) and striatum (d), expressed as percentages and folds of contralateral fluorescence intensities, respectively. Data are mean ± SEM, n = 6. *p ≤ 0.05, **p ≤ 0.01, ***p ≤ 0.001 *versus* sham; #*p* ≤ 0.05, ##*p* ≤ 0.01 *versus* vehicle; $*p* ≤ 0.05 *versus* o-NSC.

White matter injury is thought to contribute to short- and long-term behavioral deficits after stroke^25^,^26^ whereas gal-1 is known to promote axonal regeneration^27^,^28^. Thus, we examined the effects of o-NSC and s-NSC transplantation on white matter injury. To accomplish this goal, two white matter markers were assessed immunohistochemically, myelin basic protein (MBP), a marker for the integrity of myelin sheath and non-phosphorylated neurofilament H (SMI-32), a marker for demyelinated axons. Seven days after ischemia, significant decreases in MBP staining and concomitant increases in SMI-32 staining were observed in the white matter-enriched corpus callosum and striatum in vehicle-treated mice (Supplementary Fig. 2), resulting in an increased SMI-32-to-MBP ratio (Supplementary Fig. 2c,d). In mice transplanted with s-NSCs, the decreases in MBP staining and the increases in SMI-32 staining were significantly attenuated in both the corpus callosum and striatum compared to vehicle control mice (Supplementary Fig. 2c,d); in contrast, the transplantation of o-NSCs significantly enhanced MBP staining in the corpus callosum, but had no effect on SMI-32 staining in either regions. At 28 days post-injury in vehicle-treated mice, a similar pattern of changes in MBP staining and SMI32-to-MBP ratio were observed in the corpus callosum and striatum (Fig. 3a,b). In both brain regions, transplantation of o-NSCs or s-NSCs significantly elevated MBP staining and decreased the SMI32-to-MBP ratio compared to vehicle-treated mice (Fig. 3c,d). However, transplantation with s-NSCs showed significantly greater effects on the SMI-32-to-MBP ratio than transplantation with o-NSCs (Fig. 3). In sum, transplantation with s-NSCs exhibited greater protective effects against white matter injury than o-NSCs over the long term (28 days after stroke) but not in the short term (7 days after stroke).

### Gal-1 promotes the M2 phenotype in microglial cultures

**Figure 4:**
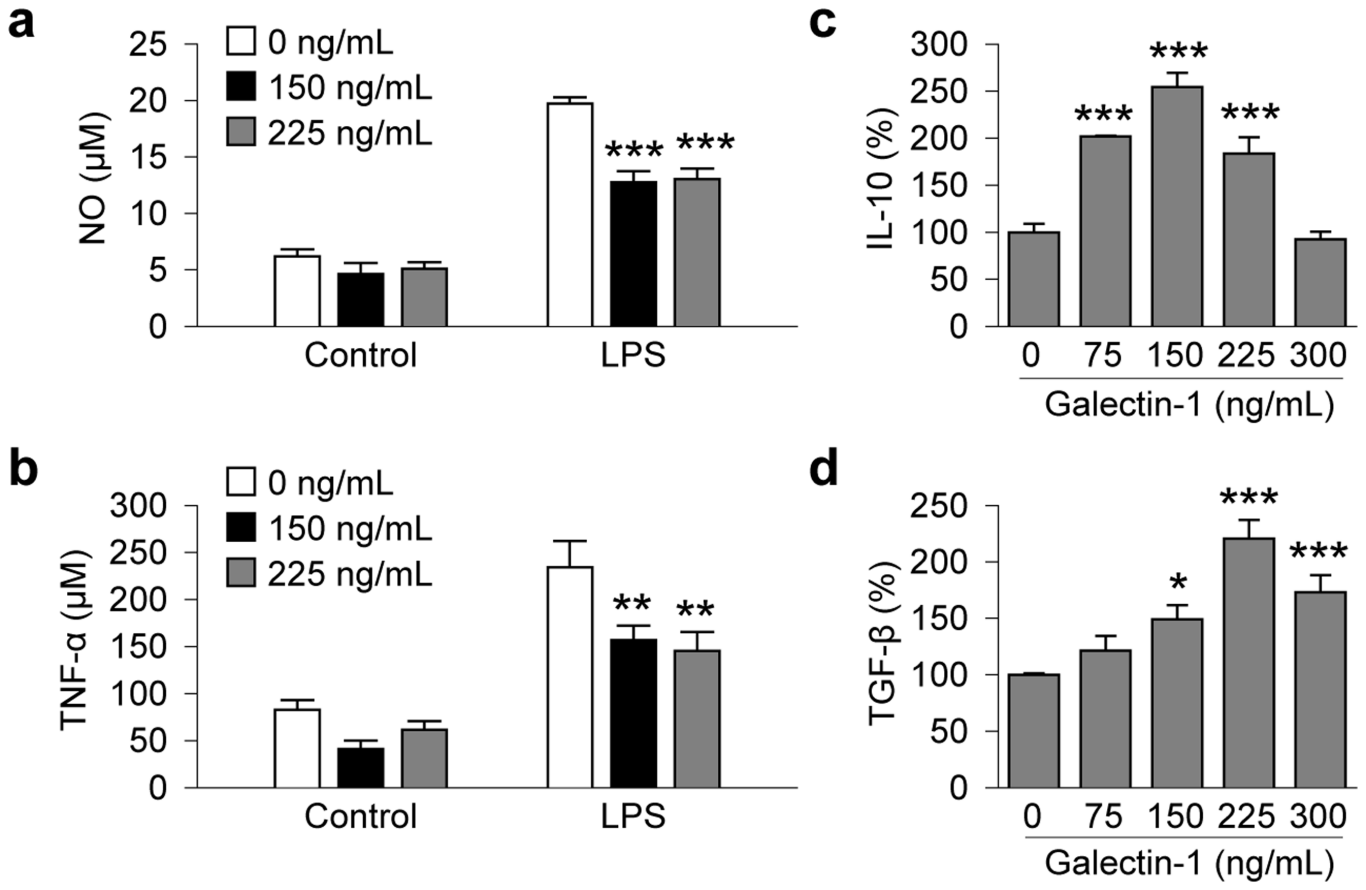
Galectin-1 inhibits NO and TNF-α production and enhances IL-10 and TGF-β gene expression in microglia challenged with LPS. BV2 microglia seeded at 5 × 10^4^/well were pretreated with galectin-1 at the indicated concentrations for 1 hr and LPS (2.5 ng/mL) or its vehicle was added. (a–b) NO and TNF-α were measured in culture medium 12 h after LPS challenge or vehicle treatment. Results are mean ± SEM, from 3 independent experiments, each conducted in triplicate. **p ≤ 0.01 and ***p ≤ 0.001 *versus* vehicle (0 ng/mL). (c–d) Quantitative RT-PCR for IL-10 and TGF-β mRNA was performed 12 h after LPS challenge following galectine-1 pre-treatment at the indicated concentrations. Data are mean ± SEM, from 3 independent experiments, each conducted in triplicate. **p ≤ 0.01 and *p ≤ 0.05 *versus* LPS only.

Thus far we had found that protection against MCAO was often more prominent in the s-NSC group than the o-NSC group, such as mitigation of tissue atrophy at 28 d (Fig. 1c), attenuation of sensorimotor deficits in the cylinder test (Fig. 2b), and improved spatial memory in the Morris water maze (Fig. 2e). The enhanced protection by s-NSCs suggests distinct juxtacrine roles of this carbohydrate binding receptor. As microglia are known targets for gal-1^29^ and microglial activation is thought to contribute to white matter injury^30^, we investigated the effects of gal-1 on the functional status of microglia. Microglia can be categorized according to two functionally distinct phenotypes: a classic phenotype (M1) and an alternative phenotype (M2). M1 microglia generally exhibit pro-inflammatory features whereas M2 microglia are associated with anti-inflammatory and neurorestorative functions after brain injuries^31^,^32^. In non-challenged control BV2 microglia-like cells, treatment with 150 or 225 ng/mL of gal-1 did not significantly alter the basal levels of NO and TNF-α (Fig. 4a,b), both of which are hallmarks of the M1 microglial phenotype. However, gal-1 at the same concentrations significantly suppressed LPS (2.5 ng/mL for 24 hours)-induced production of NO and TNF-α from BV2 cells compared to vehicle controls (Fig. 4a,b). Gal-1 also significantly enhanced the production of IL-10 and TGF-β, both hallmarks of M2 microglia, at concentrations of 75, 150, and 225 ng/mL in LPS-challenged BV2 cells (Fig. 4c,d).

**Figure 5:**
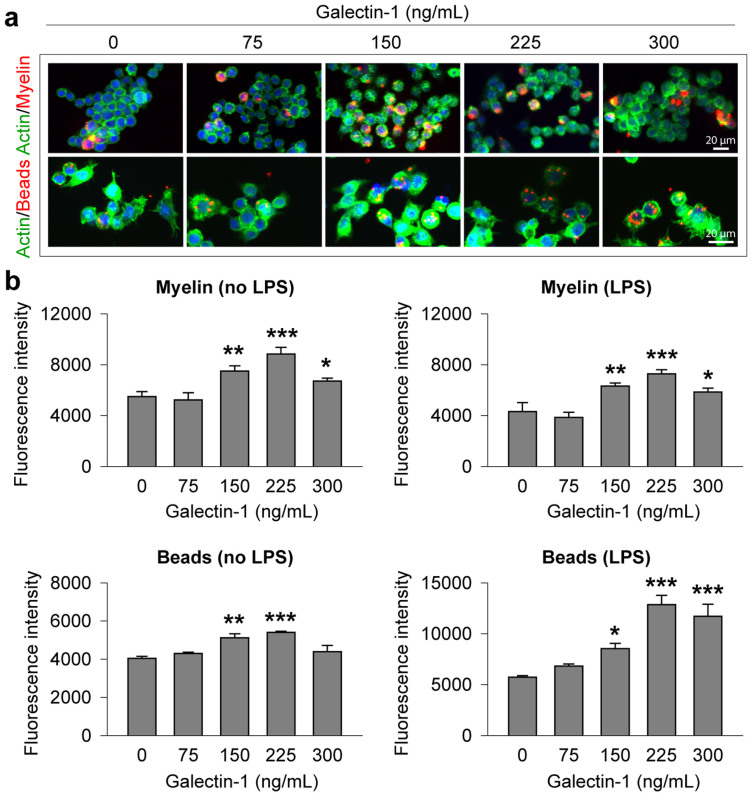
Galectin-1 enhances the phagocytic activity of BV2 microglia. BV2 microglia seeded at 5 × 10^4^/well were pretreated with galectin-1 at the indicated concentrations for 1 hr. LPS (2.5 ng/mL) or vehicle together with one of the phagocytic substrates, fluorescence-labeled myelin or microsphere beads, were added to cultures for 3 hours. (a) Representative fluorescent images show the phagocytosis of fluorescence-labeled myelin (upper panel) or microsphere beads (lower panel) into BV2 microglia after 3 hours of incubation. (b–c) Phagocytic activity of BV2 microglia was quantified by flow cytometry for fluorescence-labeled myelin (b) and microsphere beads (c), respectively, after 3 hours of incubation of either substrate with or without LPS (2.5 ng/mL). Data are mean ± SEM, from 4 independent experiments, each conducted in triplicate, *p ≤ 0.05, **p ≤ 0.01, ***p ≤ 0.001 *versus* vehicle (0 ng/mL).

Our data thus far were consistent with reports that gal-1 promotes the M1 phenotype in activated microglia/macrophages^15^,^22^,^27^. Thus, we further speculated that pre- and post-treatments with gal-1 would also enhance microglial phagocytic activity, another important function of M2 microglia. We examined the effects of galectin-1 (0–300 ng/mL) on the phagocytic activity of BV2 cells using two different types of fluorescence-conjugated phagocytic targets, red-fluorescence beads and purified myelin fused to red fluorescence protein (RFP), respectively. Pretreatment with increasing concentrations of galectin-1 in the range of 150–300 ng/mL significantly enhanced the phagocytic activity of LPS-challenged or unchallenged (vehicle-treated) BV2 cells for both phagocytic targets (Fig. 5a,b), with the exception that 300 ng/mL galectin-1 failed to increase phagocytic activity in vehicle-treated BV2 cells.

### Gal-1 primes microglia toward M2 polarization after MCAO

**Figure 6:**
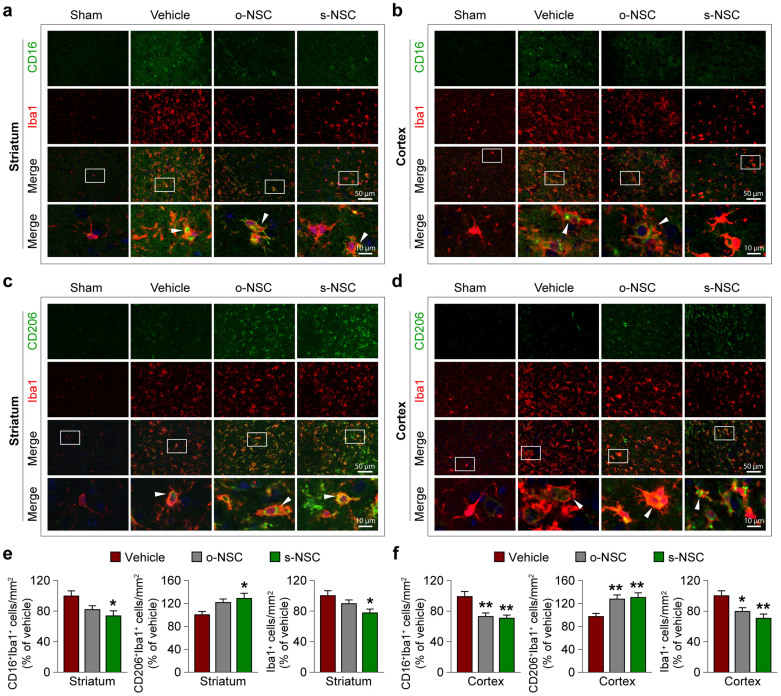
Transplantation of s-NSCs enhances microglia/macrophage polarization toward M2 phenotype after ischemia. Mice received transplantation of vehicle, o-NSCs, or s-NSCs 2 hours after MCAO or were subjected to sham surgery. Brain sections were dual-stained for CD16 and Iba-1 or CD206 and Iba-1 at 7 d after ischemia. (a–b) Representative immunofluorescence images of CD16 and Iba1 staining in the striatal (a) and cortical (b) infarct border. (c–d) Representative immunofluorescence images of CD206 and Iba1 staining in the striatal (c) and cortical (d) infarct border. (e–f) Quantification of CD16^+^/Iba-1^+^, CD206^+^/Iba-1^+^, or Iba1^+^ cells in the striatal (e) and cortical (f) infarct border. Data are expressed as percentages of cell numbers *versus* vehicle-treated stroke brains. *p ≤ 0.05 and **p ≤ 0.01 versus vehicle-treated stroke group; n = 6/group.

Finally, we performed *in vivo* experiments to determine whether s-NSC transplantation promotes M2 polarization of microglia/macrophages in mouse brains after MCAO. Consistent with the *in vitro* observations, transplantation of modified neural stem cells (either o-NSC or s-NSC) significantly decreased the numbers of CD16^+^ (M1 marker) cells but increased the numbers of CD206^+^ (M2 marker) cells in Iba1^+^ microglia/macrophages in the ischemic cortex at 7 days after ischemia (Fig. 6b,d,f). Similarly, transplantation of either o-NSCs or s-NSCs significantly decreased the numbers of CD16^+^/Iba1^+^ cells in the corpus callosum at 7 days after ischemia (Supplementary Fig. 3). In contrast, only s-NSCs decreased the numbers of CD16^+^ cells and increased the numbers of CD206^+^ cells in the ischemic striatum, where the ischemic injury was most severe in this stroke model. These results strongly suggest that transplantation of modified neural stem cells, especially the galactin-1-secreting neural stem cells, switches microglial polarization towards the beneficial M2 phenotype in the post-stroke brain, even in areas of severe ischemic injury.

## Discussion

In the present study, we transplanted two different lines of NSCs overexpressing gal-1 into the mouse brain after stroke injury and determined their effect on long-term neurological outcomes. The results demonstrate that transplantation of engineered NSCs, especially those that produce gal-1 with a secretory sequence, conferred robust neuroprotection against stroke by decreasing infarct volume, ameliorating white matter injury, and improving sensorimotor and cognitive functions for at least 28 days after stroke. Moreover, we showed that s-NSCs modulated the polarization of microglia and/or macrophages towards the alternative M2 phenotype, which is well known to facilitate neuroprotection and promote brain repair after CNS injuries^30^,^33^.

Gal-1 is encoded by the beta-galactoside-binding protein 1 gene located on chromosome 22q12, and is comprised of 135 amino acids with a molecular weight of 14.5 kDa^34^. Gal-1 is present both within cells and in the extracellular space and maximal gal-afforded cytoprotection in the present study appeared to require extracellular actions. Gal-1 does not contain any recognizable secretory signals^11^,^35^. Nevertheless, gal-1 can be found in the extracellular space, indicating that it is secreted through a yet undefined mechanism^36^. Here, we engineered a special construct by inserting a secretory signal sequence into the *gal-1* gene and stably overexpressing this construct in NSCs, thereby creating the versatile s-NSCs line. We found that s-NCSs have a markedly enhanced capacity to secrete gal-1 into cell culture media than o-NSCs containing the gal-1 gene without the secretory signal sequence.

The mechanisms underlying gal-1-mediated neuroprotection are not fully understood. Previous studies have demonstrated that gal-1 inhibits the proliferation of astrocytes, attenuates astrogliosis and down-regulates the expression of iNOS and IL-1β after cerebral ischemia^23^. On the other hand, gal-1 also induces astrocyte differentiation and differentiated astrocytes enhance production of BDNF, which has important neuroprotective roles^9^,^18^. In addition, gal-1 also affects neurogenesis in mice, as infusion of gal-1 antibodies or knockout of the *gal-1* gene decreases the number of endogenous NSCs^14^,^37^. In contrast, gal-1 delivery stimulates neurogenesis in the SVZ after ischemia^17^,^37^. However, this phenomenon may not be universal, because other studies show that gal-1 may inhibit neurogenesis in the hippocampus of C57BL/6 mice as shown by an increase in newborn cells in the dentate gyrus in *gal-1* knockout mice^38^.

Here we provide evidence that gal-1 also protects against ischemic white matter injury. It is unclear how gal-1 protects white matter, although the promotion of axonal regeneration may be one potential mechanism^27^,^28^. Once secreted from cells, gal-1 exists in either reduced or oxidized forms, depending on surrounding redox status. Oxidized gal-1 loses its lectin activity but gains an unexpected feature: promotion of neurite growth and axonal regeneration^39^. This feature of gal-1 has been observed in both peripheral nerves^40^ and the central nervous system (CNS)^41^. Gal-1 may be more potent in the CNS than in the periphery as the pM concentration range of gal-1 appears to be sufficient for this effect^12^,^42^. As oxidized gal-1 loses its stimulating effect in isolated neurons, it is assumed to affect the other cell types surrounding the axons^12^,^43^. Macrophages are one such candidate, as they release an unidentified factor that promotes axonal regeneration when stimulated by oxidized gal-1^27^. This promoting factor exhibits a molecular weight of 14 kDa but is assumed to be different from any known neurotrophic factor^27^. As oxidative stress is a prominent feature of the ischemic brain, secreted gal-1 may be rapidly oxidized after stroke and consequently promote axonal restoration and preserve white matter structure and function as a natural defense mechanism. Future studies are warranted to determine whether the long-term neuroprotective effects of s-NSCs in the stroke model involve enhanced axonal regeneration by gal-1.

The present study is the first to show that gal-l regulates the polarization of microglia *in vitro* and *in vivo* after ischemia or after exposure to LPS. Based on their stimulators and functional roles, microglia/macrophages are classified into two distinct phenotypes, M1 and M2^31^,^44^. M1 microglia/macrophages are “classically activated”, typically by LPS or interferon-γ (IFN-γ), and release destructive pro-inflammatory mediators^31^,^45^,^46^. M2 microglia/macrophages are “alternatively activated”, typically by IL-4 or IL-10 and release neuroprotective and anti-inflammatory mediators^31^,^44^,^47^. Recent studies reveal that gal-1 interacts with T cells and microglia and regulates their polarization and function. For example, stem cells that secrete gal-1 can inhibit the proliferation of T cells and their release of pro-inflammatory cytokines^21^,^24^,^36^. In particular, gal-1 decreases the release of IFN-γ and TNF-α^36^ but increases the release of IL-10 and IL-4^24^,^36^. This alteration in cytokine release patterns favors polarization toward the M2 phenotype. Recent studies have also shown that gal-1 preferentially binds M1 phenotype microglia over M2-polarized microglia^15^. Specifically, gal-1 reacts with the O-glycan of microglial CD 45, a heavily glycosylated protein with tyrosine phosphatase activity. This binding increases the retention of CD 45 on the microglial surface and the phosphatase activity of CD 45, thus pushing microglia toward the M2 phenotype^24^,^48^. M2 microglia play a critical role in the promotion of remyelination and white matter repair in demyelination disorders^49^. This property of M2 microglia may help explain the protective effects of secreted gal-1 against ischemic white matter injury in the present study. Furthermore, protection of white matter may also help preserve neuronal integrity and promote behavioral recovery. The use of microglia/macrophage-specific conditional transgenic mice will help to confirm or refute this novel hypothesis.

In summary, our data demonstrate that s-NSCs offer more profound neuroprotection against ischemic brain injury than o-NSCs. The mechanisms underlying neuroprotection are likely to be multifaceted, but may include enhanced polarization of microglia and/or macrophages toward the M2 phenotype and subsequent white matter protection. Therefore, gal-1 is a rational target for long-term neuroprotection against stroke, and transplantation of engineered NSCs secreting gal-1 into the brain after stroke is a promising therapeutic strategy. However, a limitation of the current study is the short delay (2 hours) of NSC delivery into the post-stroke brain, which may not be readily practical in clinic. In order to increase the translatability of this approach, future studies aimed at determining the maximal interval between stroke and delayed NSC transplantation are warranted.

## Methods

All animal experiments were approved by the University of Pittsburgh Institutional Animal Care and Use Committee, and performed in accordance with the *National Institutes of Health Guide for the Care and Use of Laboratory Animals*. Stroke Treatment and Academic Roundtable (STAIR) guidelines were adhered to throughout all animal studies. All measurements were performed in blinded manner wherever possible.

### *In vitro* gene construction

A pCMV6-entry vector containing mouse mgals1 (Myc-DDK-tagged) was purchased from OriGene Technologies (Rockville, MD, USA). The *gal-1* gene was cut from this plasmid with restriction enzymes AsiS1 and Mlu1 (New England BioLabs, Beverly, MA, USA), and was then inserted into the pCMV6-AC-FC-S plasmid (OriGene, Rockville, MD, USA), which contained a secretory signal sequence. The resultant plasmid contained mouse mgals1 (Myc-DDK-tagged) with a secretory signal at the N-terminal. Construction of recombinant plasmids was confirmed by PCR with products run on 1% agarose gels in TAE buffer (0.04 M Tris, 0.02 M acetic acid, 0.001 M EDTA, pH 8.0).

### *In vitro* gene transfection and cell culture

An NE-4C neural stem cell line obtained from American Type Culture Collection Center (ATCC, Manassas, VA, USA) was seeded into a 10-cm cell culture dish at a density of 5 × 10^5^ cells per well 24 hours prior to gene transfection. Cells were maintained in serum containing medium composed of minimum essential medium (MEM; Sigma-Aldrich) supplemented with 5% fetal calf serum (FCS; Invitrogen-Gibco), 4 mM glutamine (Sigma-Aldrich) and 40 μg/mL gentamycin (Chinoin), at 37°C with 5% CO_2_^50^,^51^. Cells were then transfected by the two plasmids using Lipofectamine 2000 (Invitrogen, Carlsbad, CA, USA)^52^. Two days after transfection, cells were split at a 1:10 ratio and maintained in α-MEM medium (Invitrogen) until cell colonies were formed. The colonies were selected and transferred into four six-well plates. Gal-1 was detected in the supernatant by Western blot analyses and measured with an ELISA quantification kit (R&D Systems, Minneapolis, MN, USA). Clones with no or lower amplification of gal-1 were discarded. Clones with the highest gal-1-DDK expression were cultured on a large scale. For maintenance, subconfluent NE-4C cultures were regularly split by trypsinization (0.05 w/v% trypsin in PBS) into poly-L-lysine-coated Petri dishes.

The BV2 mouse microglia cell line ICLC ATL03001 (Interlab Cell Line Collection, Banca Biologica e Cell Factory, Italy) was maintained at 37°C in Dulbecco's modified Eagle's medium supplemented with 10% fetal bovine serum in a humidified incubator with 5% CO_2_/95% air. Cells were split 1:10 when they reached confluence using trypsin/EDTA solution (Sigma)^53^,^54^.

### Assays for inflammatory factors

Culture supernatants were collected after 24 h of stimulation with LPS and treatment with galectin-1. The production of tumor necrosis factor-alpha (TNF-α) was measured with a commercial ELISA kit from R&D Systems. As an indicator of nitric oxide (NO) production, the amount of nitrite accumulated in culture supernatants was determined with a colorimetric assay using the Griess reagent (1% sulfanilamide, 2.5% H_3_PO_4_, 0.1% N-(1-naphthyl) ethylenediamine dihydrochloride), as described previously^55^.

### Real-time RT-PCR

Total RNA was isolated from cell culture samples using the RNeasy Mini Kit according to the manufacturer's instructions (Qiagen, Valencia, CA, USA)^31^, and 5 μg was used to synthesize the first strand of cDNA using random hexamer primers and the Superscript First-Strand Synthesis System for RT-PCR (Invitrogen). PCR was performed using SYBR green PCR Master Mix (Applied Biosystems, Foster City, CA). The forward and reverse primers of TGF-beta were TGG CGT TAC CTT GGT AAC and GGT GTT GAG CCC TTT CCA, respectively. The forward and reverse primers of IL-10 were GGC TCA GCA CTG CTA TGA TGC C and AGC ATG TGG GTC TGG CTG ACT G, respectively. The cycle time (Ct) values of the genes of interest were first normalized with GAPDH of the same sample, and then the gene expression levels in vehicle- and galectin-1-treated groups were calculated and expressed as percentage changes versus vehicle (0 ng/mL gal-1).

### Phagocytosis assay

BV2 mouse microglia cells were incubated with gal-1 or vehicle for 24 hours. Cells were then incubated in the presence or absence of Cy-3-labeled myelin (0.5 μg/mL) for an additional 6 hours. To quench the signal from extracellular or outer plasma membrane-associated microspheres, the medium was removed and the cells were thoroughly rinsed with 0.25 mg/mL Trypan blue in phosphate-buffered saline (PBS) for 1 min. Cells were lysed using PBST (1% Triton in PBS). Intracellular fluorescence was then measured using a fluorescence plate reader at 535 nm excitation and 575 nm emission. For image analysis of phagocytotic structures, microglia were plated at 1 × 10^5^ cells/well into 8-well chamber slides (Nunc). Cy3-labeled myelin was then added as described above. Cells were subsequently rinsed with PBS and fixed in 4% paraformaldehyde. AlexaFluor488 phalloidin (1:50, Invitrogen) was added to these fixed cultures for 1 hour at room temperature in the dark. Images were captured with an Olympus confocal microscope by a blinded observer.

### Western blot analysis

Cell lysates were centrifuged at 14,000 × g at 4°C for 30 minutes. Supernatants were then collected and total protein was determined by the bicinchoninic acid assay^31^. Equal amounts of protein were resolved by standard sodium dodecyl sulfate-polyacrylamide gel electrophoresis and transferred to nitrocellulose membranes. After blocking membranes for 1 h in 5% nonfat milk dissolved in Tris-buffered saline solution, membranes were incubated overnight in primary antibodies at 4°C. Membranes were then subjected to 3 consecutive washes with Tris-buffered saline for 5 minutes each, followed by incubation with goat anti-rabbit immunoglobulin G conjugated to horseradish peroxidase (HRP, Pierce Biotechnology Inc., Rockford, IL, USA) at room temperature for 1 h. Membranes were scanned and quantified by Scion Image (Scion, Frederick, MD, USA), and protein levels were expressed as a fraction of β-actin in the same lane.

### Ischemia model

Male C57BL/6 mice (Jackson Laboratory) were used for all experiments. Transient focal cerebral ischemia was induced by intraluminal occlusion of the left middle cerebral artery for 60 minutes as described previously^56^,^57^. Cortical cerebral blood flow (CBF) was measured using laser Doppler flowmetry to confirm the induction of cerebral ischemia after MCAO^56^,^57^. Animals that did not show a CBF reduction of at least 75% over baseline levels were excluded (<10% of stroke animals) from further experimentation. Animals that died after ischemia induction were also excluded. Rectal temperature was maintained at 37 ± 0.5°C during the ischemic period. At 2 hours after MCAO, animals were randomly assigned to vehicle, o-NSC, or s-NSC groups. Investigators were blinded to treatment groups during cell transplantation or vehicle injection and during all outcome assessments.

### NSC transplantation

Two hours after MCAO, mice were subjected to transplantation of various types of NSCs into the ipsilateral hemisphere^16^,^19^. Animals were anesthetized with 1.5% isoflurane and placed in a stereotaxic frame. Using Hamilton syringes, a 3-μl aliquot of NSC suspension (50,000 cells) was injected into the left striatum (AP: 0.5 mm, ML: 1.9 mm, DV: 3.5 mm) and an identical aliquot was injected into the left cortex (AP: 0.38 mm, ML: 1.0 mm, DV: 0.8 mm) over the course of 10 min. The syringe was left in place for an additional 5 min to allow diffusion from the tip.

### Rotarod test

The rotarod test was used to evaluate motor coordination by testing the ability of mice to remain on a revolving rod. Mice were pre-trained for three consecutive days before MCAO in an accelerated rotarod training paradigm^56^,^58^ and tests were performed 3 days after MCAO. The revolving speed was increased from 0 to 30 rpm and maintained at this final speed for 5 min. The trial ended if the animal fell off the rungs or gripped the device and spun around for 2 consecutive revolutions without attempting to walk on the rungs. The latency to fall or spin around on the rungs was quantified.

### Cylinder test

The cylinder test was employed to assess forelimb use and asymmetries in postural weight support during exploratory activity^59^. Mice were pre-trained on the cylinder test before ischemia and the test was performed 3 days after ischemia. During the test, mice were placed in a transparent Plexiglas cylinder (20 cm high, 5 cm diameter). As the animals reared to explore the environment, the numbers of bilateral, ipsilateral and contralateral paw placements were recorded and analyzed off-line. The relative proportion of left (ipsilateral) forepaw contacts was calculated as: (left-right)/(left + right + both) × 100^60^.

### Morris water maze test

Cognitive function was determined using a water maze task as described previously^32^,^56^,^61^ with minor modifications. Briefly, the water maze was a black-colored circular pool filled with water (21–23°C) and situated in a room with salient visual cues. Each mouse received 3 consecutive trials (with randomly assigned starting positions) per day to locate the platform submerged 2 cm beneath the water surface. They were allowed 60 s to locate the platform, which was kept in a constant location. The mean escape latency per day was recorded for each animal and used in the statistical analysis as learning capacity. One day after the final acquisition training session, all of the mice performed a probe test with the escape platform removed. The animals were placed into the pool in the location most distal to the target quadrant (with the platform removed). The percent of time spent in the target quadrant was recorded and interpreted as spatial memory. Acquisition of spatial learning was performed during post-operative days 24–28 for five consecutive days^62^.

### Immunohistochemistry

At the indicated time points, mice were sacrificed by perfusion and brain sections were prepared for immunostaining using previously reported methods^31^,^56^. Similarly, cultures were fixed at the indicated times and prepared for immunostaining. The following primary antibodies were used: anti-MAP2 (1:200; Santa Cruz Biotechnology), anti-β-actin (1:8000; Millipore), anti-gal-1 (1:100; R&D), anti-MBP (1:500; Abcam), SMI-32 (1:300; Millipore), anti-Iba1 (1:1000; Wako), anti-DDK (1:100; Millipore), anti-GFAP (1:500, Dakocytomation), anti-CD16 (1:500, BD Biosciences), and anti-CD206 (1: 500, R&D Systems). DAPI was used as the nuclear counterstain. Immunofluorescence was visualized on a confocal fluorescent microscope and analyzed by a blinded observer using the Image J analysis system.

### Statistical analyses

All data are presented as mean ± standard error of the mean (SEM) from at least three independent *in vitro* experiments, each conducted in triplicate, or at least 6 animals per group *in vivo*. Differences in infarction volumes, rotarod, cylinder, and Morris water maze tests were analyzed using ANOVA followed by *post hoc* Bonferroni tests. P < 0.05 was considered statistically significant.

## Author Contributions

J.C., L.C. and A.K.F.L. designed the research. J.X., J.W., Y.S., Y.W. and H.P. performed the research. F.Z., Y.S., X.Y., L.C. and J.C. analyzed the data. F.Z., A.K.F.L., R.K.L. and J.C. wrote the manuscript. All authors reviewed and edited the manuscript.

## Supplementary Material

Supplementary Information Supplementary Information
