# Supplementary material for: Galectin-1-secreting neural stem cells elicit long-term neuroprotection against ischemic brain injury
Source: Sci Rep. 2015 Apr 10;5:9621. doi: 10.1038/srep09621 (PMC4392363; doi:10.1038/srep09621)
Supplement: Supplementary Information [file srep09621-s1.doc]

Supplementary Information

**Galectin-1-secreting neural stem cells elicit long-term neuroprotection against ischemic brain injury**

Jiayin Wang, Jinchao Xia, Feng Zhang, Yejie Shi, Yun Wu, Hongjian Pu,

Anthony K.F. Liou, Rehana K. Leak, Xinguang Yu, Ling Chen, and Jun Chen

**
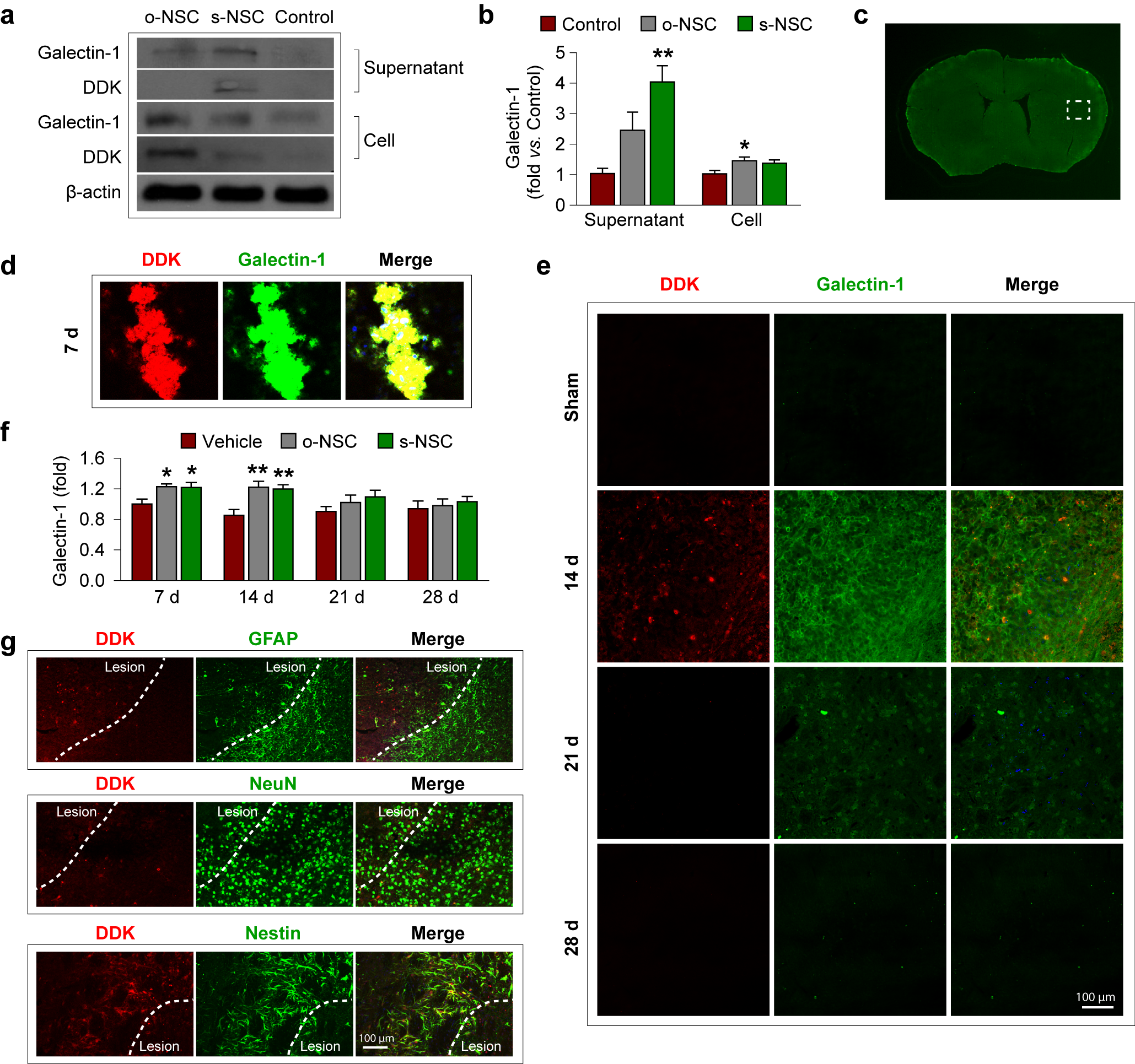
**

**Supplementary Figure 1. Modified neural stem cells secrete galectin-1 and remain viable 14 days after transplantation into post-ischemic brains.** (**a-b**) Representative Western blots and semi-quantitative analysis of galectin-1 levels in the supernatants and cell lysates, respectively, from the NE-4C neural stem cells stably overexpressing the wild type galectin-1 (o-NSCs) or the secretory galectin-1 (s-NSCs) or the non-transfected NSCs (control cells) for 24 hours. Data are mean ± SEM, n=3. **p*≤0.05, ***p*≤0.01 *versus* control cells. (**c-e**) The fate of transplanted neural stem cells in the brain after stroke. Two 3-μl aliquots of s-NSCs (50,000 cells/aliquot) were infused into the cortex and striatum, respectively, at 2 hours after 60 min of MCAO. Double-label immunefluorescent staining for DDK and galectin-1 was performed at 14, 21 and 28 d after cell transplantation. Representative images are presented: the box in (**c**) depicts the approximate brain region where the representative images were taken; the images in (**d**) show the double positive staining for DDK and galectin-1 in a cluster of transplanted cells in brain at 7 d after MCAO. The images in (**e**) show many double positive cells in brain at 14 d after MCAO, but few cells at 21 or 28 d after MCAO. (**f**) Galectin-1 levels in the striatum were measured using ELISA at 7, 14, 21, and 28 d after s-NSC transplantation. The levels are expressed as fold increases versus the vehicle controls at 7 d after cell transplantation. n=5/group, **p*≤0.05, ***p*≤0.01 *versus* vehicle group at 7 d. (**g**) Differentiation of transplanted s-NSCs in the brain at 14 d after cell transplantation. The NSCs were co-stained for DDK (*red*) with GFAP (astrocyte marker), NeuN (neuronal marker) or Nestin (stem cell marker) in *green*. Whereas most transplanted s-NSCs showed the expected co-localization with nestin, less than 10% the transplanted cells expressed NeuN or GFAP.


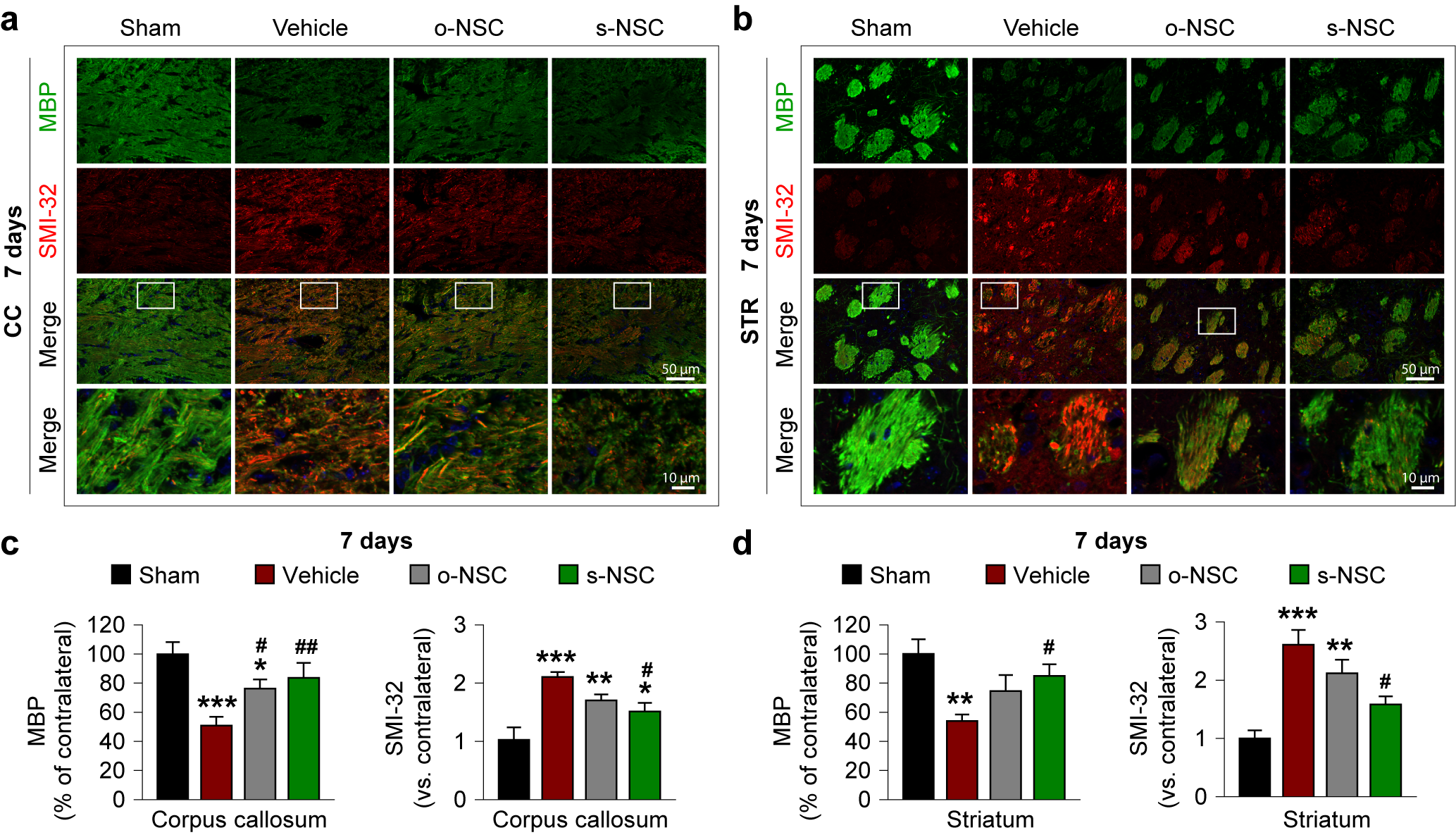


**Supplementary Figure 2. Transplantation of s-NSCs improves white matter integrity 7 days after ischemia.** Mice received transplantation of o-NSCs or s-NSCs 2 hours after MCAO. Brain sections were dual-stained for myelin basic protein (MBP) and non-phosphorylated neurofilament H (SMI-32) on Day 7 after ischemia. (**a-b**) Representative immunofluorescent images of MBP and SMI-32 staining in the corpus callosum (CC, **a**) and striatum (STR, **b**) after sham surgery or MCAO followed by transplantation of vehicle, o-NSCs, or s-NSCs. (**c-d**) Quantification of MBP and SMI-32 immunofluorescence in the corpus callosum (**c**) and striatum (**d**), expressed as percentages and folds of contralateral fluorescence intensities, respectively. Data are mean ± SEM, n=6. **p*≤0.05, ***p*≤0.01, ****p*≤0.001 *versus* sham; #*p*≤0.05, ##*p*≤0.01 *versus* vehicle.

##
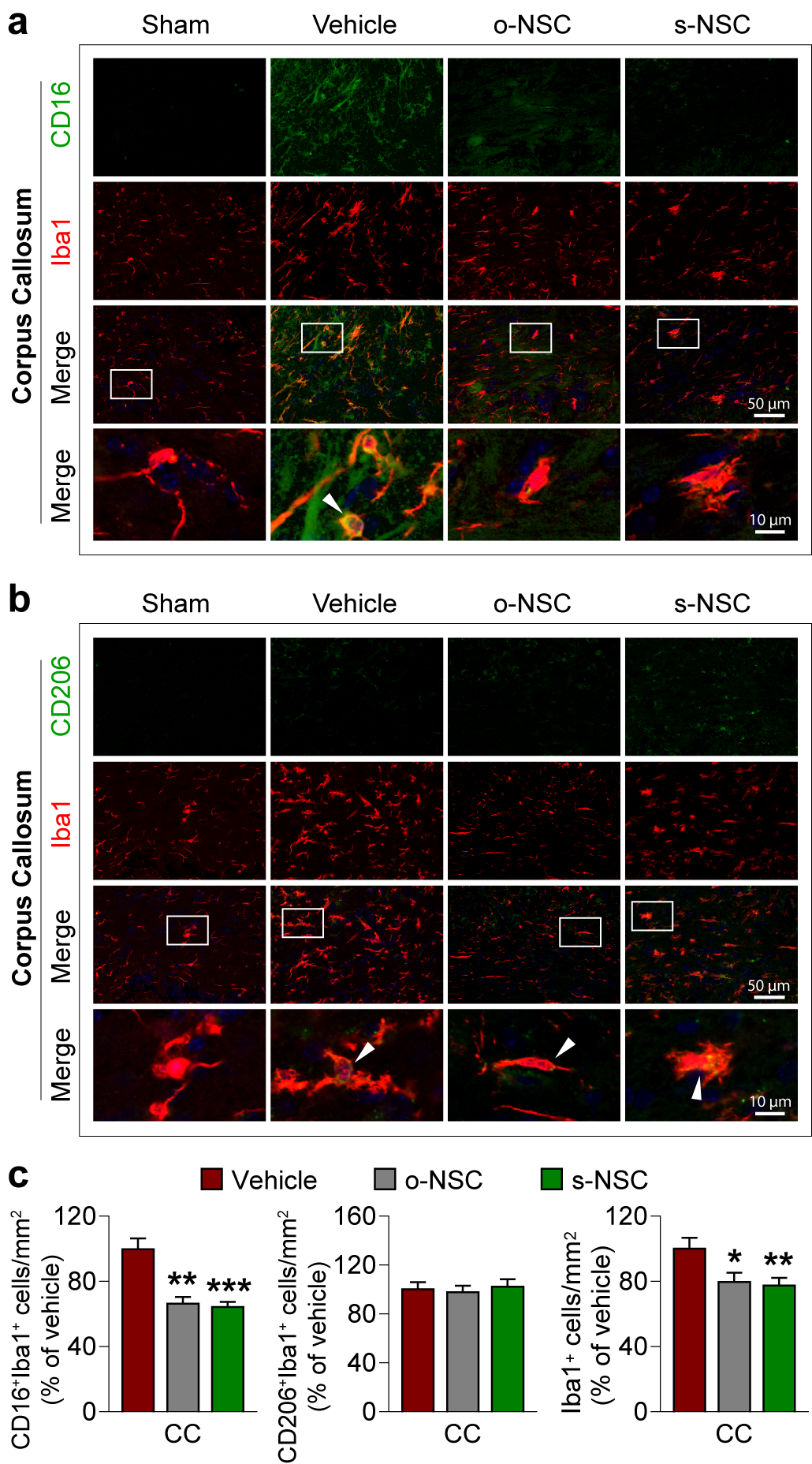


## Supplementary Figure 3. Transplantation of s-NSCs suppresses microglia/macrophage M1 polarization in the corpus callosum after ischemia. Mice received transplantation of vehicle, o-NSCs, or s-NSCs 2 hours after MCAO or were subjected to sham surgery. Brain sections were dual-stained for CD16 and Iba1 or CD206 and Iba-1 at 7 d after ischemia. (a-b) Representative double-label immunofluorescence images of CD16 and Iba1 staining (a) and CD206 and Iba1 staining (b) in the corpus callosum. The bottom panels in (a) and (b) are high power images of the regions indicated in the boxes. The *arrowhead* in the bottom panel in (a) points to a CD16^+^/Iba1^+^ cell; the *arrowheads* in the bottom panel in (b) point to Iba1^+^ cells with little CD206 staining. (c) Quantification of CD16^+^/Iba1^+^, CD206^+^/Iba1^+^, or Iba1^+^ cells in the corpus callosum. Data are expressed as percentages of cell numbers *versus* vehicle-treated stroke brains. **p*≤0.05 and ***p*≤0.01 *versus* vehicle-treated stroke group; n=6/group.
